# Perspective: Vitamin D Deficiency Relationship to Initiation of Diseases

**DOI:** 10.3390/nu17172900

**Published:** 2025-09-08

**Authors:** David R. Fraser

**Affiliations:** Sydney School of Veterinary Science, Faculty of Science, The University of Sydney, Sydney, NSW 2006, Australia; david.fraser@sydney.edu.au

**Keywords:** vitamin D deficiency, solar UV-B radiation, skeletal muscle, intracrine, autocrine, paracrine functions of 1,25(OH)_2_D, vitamin D-binding protein, randomized clinical trials

## Abstract

Vitamin D is converted to a steroid hormone by 25-hydroxylation in the liver and then by 1-hydroxylation in the kidney to produce the circulating hormone 1,25-dihydroxy vitamin D [1,25(OH_2_D]. This hormone then functions in cells of the intestinal mucosa and in bone to maintain whole-body calcium homeostasis. Classical vitamin D deficiency thus results in defective calcium homeostasis. Yet vitamin D deficiency is often reported in people with various diseases not associated with whole-body calcium homeostasis. Because of these associations with vitamin D deficiency, clinical trials have been undertaken to determine whether raising vitamin D status could be an effective treatment for such diseases. However, the results of such clinical trials have largely been inconclusive. The steroidal autocrine or paracrine role of locally produced 1,25(OH)_2_D in many nonrenal cells throughout the body is protective against a range of pathological changes. In vitamin D deficiency such protection becomes defective. A disease process may thus be initiated, and then progress, while vitamin D status is inadequate, as in the months of winter in temperate regions of the world. The subsequent correction of vitamin D deficiency may no longer be able to protect patients when the disease process has already become established. To maintain the many protective roles of vitamin D against disease, it is important that public health strategies aim to maintain adequate vitamin D status throughout the year.

## 1. Introduction

Over 50 years ago it became apparent that the concentration of 25-hydroxyvitamin D [25(OH)D] in blood plasma gave an effective indication of vitamin D status [1]. However, consensus views of the 25(OH)D concentrations indicating either (a) adequate vitamin D status, (b) developing deficiency or (c) actual deficiency are still not universally agreed upon [2,3]. The difficulty of defining adequate status is compounded by the inevitable variation in vitamin D status caused by seasonal changes in solar ultraviolet light intensity, which converts 7-dehydrocholesterol in skin into pre-vitamin D_3_ [4]. Even if the serum concentration of 25(OH)D declines in the months of winter, it may still be above the levels that various expert committees have defined as states of vitamin D inadequacy or deficiency [2,3].

It is now more than 100 years since clues were found about a chemical substance in some animal fats that, when fed to dogs, prevented or treated the disease of experimental rickets [5,6]. This finding was made in the era when the dietary vitamin micronutrients were being discovered and identified, so it was inevitable that this newly described substance was defined as a micronutrient and named as ‘vitamin D’ [7]. However, if the findings of Huldschinsky [8] and of Chick et al. [9], that exposure of skin to ultraviolet light cured the bone disease of rickets, had been published before the nutritional studies that led to the antirachitic chemical being defined as a micronutrient, the endogenous supply of this substance from skin exposed to the sun might have become the physiologically accepted source.

It is now fully understood that vitamin D is metabolized by hydroxylase enzymes, first in the liver to produce 25(OH)D and then, after transport in the circulation, to the trihydroxy secosterol, 1,25-dihydroxyvitamin D, in the proximal tubule cells of the kidney [10]. Before the discovery and classification of the parent vitamin D as a nutrient, the very few foods that contain traces of this substance [11] would not have been sufficient to prevent widespread vitamin D deficiency in humans and most other terrestrial animals. Nevertheless, because of its definition as one of the micronutrients, strategies to prevent vitamin D deficiency in populations have inevitably been based on foods fortified with vitamin D or on oral vitamin D supplements [4,12,13,14].

Yet population surveys continue to show that vitamin D status declines over the winter months, even though food fortification and advice on oral vitamin D supplements are population strategies for preventing vitamin D deficiency in temperate-region countries [11,12,13,14]. This seasonal variation in vitamin D status is not just a characteristic of human populations but is also seen in wild animals with habitats at temperate latitudes. A decline in serum 25(OH)D concentration in winter has been found in wild Soay sheep [15], llamas and alpacas [16], wild boars [17], white-tailed deer [18], wild rabbits [19] and wild ground squirrels [20]. Hence a winter decrease in vitamin D status, because the low intensity of solar UV-B radiation is producing less vitamin D in skin, has had a persistent environmental impact on the evolutionary adaptation of diurnal terrestrial vertebrates. If humans are unable to gain enough vitamin D by exposure of their skin to solar UV-B radiation in summer, then they may not be able to maintain adequate vitamin D status during the subsequent period of winter, unless alternative supplies are taken by mouth.

One mechanism to avoid harmful consequences of falling vitamin D status in winter would be the evolutionary development of a storage mechanism for the 25(OH)D being generated during exposure to the high intensity UV-B radiation in summer. Yet no tissue or organ store of either vitamin D or of 25(OH)D has been discovered. There have been claims that vitamin D found in the lipid droplets of adipose tissue cells is a functional store [21] to be released as vitamin D status falls. Yet no mechanism has been found which would release vitamin D from adipocytes, other than during general fat mobilization when there was a need to obtain the stored energy of fatty acids in adipose tissue [22].

The circulating vitamin D-binding protein [DBP] has an affinity for vitamin D which is lower than that both for 25(OH)D and for the vitamin D hormone, 1,25(OH)_2_D] [23]. With adequate vitamin D status only 2–3% of the vitamin D-binding sites of the total plasma DBP are occupied by vitamin D or its metabolites. So theoretically, this vast excess of apo-DBP in the circulation could attract traces of vitamin D to diffuse from the adipocyte fat droplets. But no mechanism has been identified where such a process would be enhanced when vitamin D status is low.

However, there is a mechanism, dependent on DBP, that does result in a prolonged residence time of 25(OH)D in blood plasma, when vitamin D status is declining. That mechanism is the repeated uptake and release of 25(OH)D by the large body mass of skeletal muscle cells that results in an increased half-life of circulating 25(OH)D [24,25,26]. This process is the uptake of DBP along with other plasma proteins into muscle cells mediated by membrane megalin/cubilin endocytosis. DBP has an actin-affinity binding site [23], so when it is internalized into muscle cells it binds to cytoplasmic actin and then collects the traces of free 25(OH)D that diffuse into those cells. DBP and other plasma proteins in the cytoplasm of myocytes then undergo proteolytic degradation to release amino acids that are used for protein synthesis or as energy substrates for skeletal muscle contraction. This releases the DBP-bound 25(OH)D which then diffuses from the cell and binds again to the plentiful apo-DBP in the circulation. The repeated uptake and release of 25(OH)D by skeletal muscle cells prolongs the half-life of 25(OH)D in blood. The endocrine enhancement of DBP uptake by parathyroid hormone and possibly other hormones [27] increases the efficiency of this muscle conservation mechanism.

A key stimulus for prolonging the residence time of 25(OH)D in blood is when muscle undergoes regular physical exercise [28,29,30]. Therefore, although there is no organ or tissue where vitamin D or 25(OH)D can be deposited and then mobilized when vitamin D status is falling, the repeated passage of 25(OH)D into and out of skeletal muscle cells explains its prolonged half-life in the circulation for as long as 12–13 weeks [31]. Animals in the wild, seeking food, are physically active throughout their lives and can thus maintain adequate 25(OH)D levels in winter by its conservation in the DBP-muscle mechanism. Humans, on the other hand, may become functionally vitamin D deficient during winter, because of inadequate skin exposure during the months of summer, along with the use of sunscreen creams that block solar UV-B radiation on skin, as well as a sedentary lifestyle which impairs the skeletal muscle conservation of 25(OH)D in the months of winter.

## 2. Endocrine Role of 1,25(OH)_2_D

With the discovery of 1,25(OH)_2_D, produced in the kidney, as the functional form of vitamin D, its endocrine role in calcium homeostasis [32] was then considered to explain the whole biological purpose of vitamin D. However, it subsequently became apparent that the vitamin D receptor (VDR) mediating the cell functions of 1,25(OH)_2_D is widely distributed in cells of different tissues and organs that are not involved in calcium homeostasis [33]. Furthermore, it was discovered that the 1-hydroxylase enzyme, CYP27B1, producing 1,25(OH)_2_D, is located also in many nonrenal cells [34] where vitamin D has intracrine, autocrine or paracrine hormonal functions unrelated to whole-body calcium homeostasis.

The action of locally produced 1,25(OH)_2_D may still have a role in cell calcium homeostasis in a wide variety of cells by modifying calcium ion channels in mitochondria [35,36]. However, it is unlikely that this role in cellular calcium ion flux explains the full range of regulatory functions throughout the body of locally produced 1,25(OH)_2_D. The activation of a very large number and wide range of genes by 1,25(OH)_2_D indicates many different cell functions, apart from calcium ion transport, are under vitamin D control [37].

Although the hormonal form of vitamin D, 1,25(OH)_2_D, is produced by both renal proximal tubule cells and many other cell types, the regulation of its production is very different between the renal and the multiple extrarenal sites. In the renal proximal tubule cells, the activity of the 1-hydroxylase CYP27B1 enzyme is tightly regulated. It is upregulated by parathyroid hormone and downregulated by fibroblast growth factor-23 (FGF-23), as well as its activity being also modified by calcium and phosphate plasma concentrations and by 1,25(OH)_2_D itself, according to the requirements for generating 1,25(OH)_2_D to maintain calcium homeostasis [38,39].

In comparison, the activity of the extrarenal 1-hydroxylases is largely determined by the supply of the substrate 25(OH)D [40,41]. Most of the 25(OH)D in the circulation is bound tightly to DBP with some less-tightly bound to plasma albumin. At the normal DBP concentration in human plasma of about 6 µmol/L, and with 25(OH)D concentrations of 50–100 nmol/L, only about 0.1% of the total plasma 25(OH)D is not bound to a plasma protein [23] and is able to diffuse freely like other free steroids into cells [42]. When total plasma 25(OH)D concentration declines, as in the months of winter, with the decline of vitamin D_3_ production in skin, the supply of free 25(OH)D to extrarenal cells for 1-hydroxylation also falls. Hence the production of 1,25(OH)_2_D for the intracrine, autocrine or paracrine regulation of cell function is critically dependent on an adequate concentration of plasma 25(OH)D to ensure that the non-calcium homeostasis functions of vitamin D are maintained. This contrasts with the renal production of 1,25(OH)_2_D, which continues to be secreted in quantities required for calcium homeostasis when vitamin D status is falling. The explanation for this relative independence to changes in plasma 25(OH)D concentration is that the renal proximal tubule cells have megalin/cubilin endocytosis of plasma DBP which enables the retention and accumulation of substrate 25(OH)D that diffuses into those cells [43].

## 3. Vitamin D Deficiency and Disease

The discovery of the VDR for 1,25(OH)_2_D in many previously unsuspected cell types, along with the discovery of CYP27B1 1-hydroxylase synthesis of 1,25(OH)_2_D from 25(OH)D in many nonrenal cells, indicated that vitamin D had functions unrelated to calcium homeostasis. From this developed the idea that vitamin D deficiency, often found in people with various diseases, may have been a factor in the etiology of those diseases. Surveys demonstrated that low vitamin D status or vitamin D deficiency, defined by low serum concentrations of 25(OH)D, were found in various types of cancer [44,45], cardiovascular diseases [46], neurodegenerative diseases [47], diabetes [48] and infectious diseases [49], so the concept developed that a specific treatment for such diseases could be the correction of vitamin D deficiency by oral vitamin D supplementation.

However, when randomized clinical trials have been undertaken to test the hypothesis that vitamin D deficiency was a causative factor in such diseases and that correction of the deficiency would be an effective treatment, the results have been disappointingly inconclusive [50,51,52,53]. The only clinical trials of oral vitamin D supplementation to improve vitamin D status that were clearly shown to be effective were in the treatment or prevention of acute respiratory tract infections [49,51,54]. In many other diseases where vitamin D deficiency has been reported, the effects of the disease could well have limited the vitamin D supply, by reducing opportunities for skin exposure to the sun in summer as well as, because of immobility, reducing the ability of inactive skeletal muscle to conserve 25(OH)D in the circulation [26].

Unlike the other fat-soluble vitamins A, E and K, vitamin D is the precursor of an endocrine steroid, 1,25(OH)_2_D. If there is a deficiency of this endocrine product of vitamin D there is the risk that many of its regulatory functions, particularly of the intracrine, autocrine or paracrine types, will become defective. The other fat-soluble vitamins, and indeed also the water-soluble vitamins, all have biochemical or metabolic functions that could become defective in deficiencies of those micronutrients. But, in comparison to vitamin D deficiency, the metabolic or biochemical defects of the organic micronutrient deficiencies can be simply corrected when an adequate dietary input is provided. The affected cells have their micronutrient-linked functions restored and the clinical signs of the deficiency disease are then resolved. Although restoration of the vitamin D hormonal multicellular functions by supplying more vitamin D may enable those functions to become fully operational again, the consequences of the defective endocrine vitamin D function that developed during the time of deficiency may have become independent of further vitamin D endocrine action.

For example, this could apply to the multiple functions of 1,25(OH)_2_D in cells of the immune system. Although the classical understanding of the role of vitamin D is to maintain adequate absorption of dietary calcium and phosphate and the development and maintenance of the bony skeletal system [5,6,7,8,9], it is likely that vitamin D had an endocrine role in immune cell function before its role in calcium homeostasis had evolved. The soft-bodied vertebrate sea lamprey *Petromyzon marinus*, with no calcified tissues, is estimated to have evolved some 550 million years ago [55]. Yet these jawless fish have both 1,25(OH)_2_D in their blood and the VDR in their cells [56]. The many immunomodulatory actions of cells of the immune system in protecting against microbial infection, in sensitizing cells with abnormal function to undergo apoptosis, in promoting cell-to-cell adhesion and maintaining tight junctions between epithelial cells and in inhibiting the proliferation of neoplastic cells are all aspects of the continuous intracrine or paracrine action of locally produced 1,25(OH)_2_D [45,49,57,58,59,60]. Epidemiological studies have shown that populations living at low latitudes of high solar UV-B intensity throughout the year have a lower incidence of internal cancers, although skin cancer rates are higher [61]. People in such populations are likely to maintain adequate vitamin D status because of the persistent production of vitamin D_3_ in skin by their ongoing daily exposure to solar UV-B radiation. Therefore, the avoidance of seasonal inadequate vitamin D status in the months of winter would ensure that the autocrine hormonal role of 1,25(OH)_2_D was fully functional.

Although a considerable proportion of populations at temperate latitudes of the world have low vitamin D status in winter, many deficient individuals do not develop any of the diseases apparently linked to very low vitamin D status [60]. This indicates that vitamin D deficiency alone does not inevitably initiate a disease which is epidemiologically associated with that deficiency. Rather, the diminished autocrine or paracrine roles of 1,25(OH)_2_D, particularly in the immune system cells, would allow harmful agents or aberrant physiological processes to initiate pathological changes. If the vitamin D deficiency continues, because of prolonged seasonal low solar UV-B radiation [62], those pathological changes may no longer be susceptible to the protective intracrine cell action of 1,25(OH)_2_D, so the pathology develops into a systemic disease. Subsequent restoration of adequate vitamin D status may not be effective in treating the disease because the local intracrine actions of 1,25(OH)_2_D are directed at preventing disease initiation rather than being a therapeutic agent against established diseases. Chronic diseases with higher prevalence in populations in temperate regions such as autoimmune diseases [63] and developmental psychopathological diseases [64] may thus have been initiated at a time of inadequate vitamin D status but be unresponsive when vitamin D status improves.

## 4. Conclusions

A vulnerability to various disease initiators with seasonal vitamin D deficiency would emphasize the importance of maintaining adequate vitamin D status throughout the year. Hence it would be wise for public health education to highlight the risk of periodic low vitamin D status during the months of winter. This would apply particularly to those with limited exposure to the sun in summer and with sedentary lifestyles that impair the ability of skeletal muscle to conserve 25(OH)D when there is an inadequate vitamin D supply.
